# Bluetongue Virus Serotypes 1 and 4 in Red Deer, Spain

**DOI:** 10.3201/eid1603.090626

**Published:** 2010-03

**Authors:** Belén Rodríguez-Sánchez, Christian Gortázar, Francisco Ruiz-Fons, José M. Sánchez-Vizcaíno

**Affiliations:** Universidad Complutense de Madrid, Madrid, Spain (B. Rodríguez-Sánchez, J.M. Sánchez-Vizcaíno); Instituto de Investigación en Recursos Cinegéticos IREC, Ciudad Real, Spain (C. Gortázar); Department of Animal Health, Berreaga, Derio, Bizkaia, Spain (F. Ruiz-Fons)

**Keywords:** Antibodies, RT-PCR, serotypes 1 and 4, wildlife reservoir, wild ruminants, Bluetongue virus, red deer, viruses, Spain, dispatch

## Abstract

We studied the potential of red deer as bluetongue maintenance hosts and sentinels. Deer maintained detectable bluetongue virus (BTV) serotype 4 RNA for 1 year after the virus was cleared from livestock. However, the virus was not transmitted to yearlings. BTV serotype 1 RNA was detected in red deer immediately after its first detection in cattle.

Bluetongue (BT) is a vector-borne disease caused by a virus belonging to the genus *Orbivirus*, with 24 known serotypes (*1*). Since 2000, four of these seroptypes have been found in Spain on 5 occasions: 1) Bluetongue virus serotype 2 (BTV-2) was detected in 2000 in the Balearic Islands, 2) BTV-4 was detected in 2003 in the Balearic Islands, 3) a different BTV-4 strain was detected in 2004 in southern Spain, 4) BTV-1 was detected for the first time in 2007 in Spain, and 5) BTV-8 was detected in 2008 in Spain after it entered through the border with France. In livestock, BTV-4 was detected for the last time in November 2006, and the country was declared free of BTV-4 in March 2009 by the European Union Standing Committee on the Food Chain and Animal Health (http://rasve.mapa.es/Publica/Noticias/Ficheros/Informelibreserotipo4final.pdf). Currently, all of Spain is considered a restriction zone for BTV-1 and -8.

Sheep are considered the most vulnerable species for BT, but other ruminants are known to play a major role in BT epidemiology. The role of wild ruminants in the spreading and persistence of the virus has only begun to be elucidated. Several studies have reported the presence of either BTV antibodies (*2*,*3*) or the virus (*4*) in red deer (*Cervus elaphus*), roe deer (*Capreolus capreolus*), mouflons (*Ovis aries*), and several other wild bovids and cervids (*2*,*5*). The presence of BTV and BTV-specific antibodies in wild species underscores the role of these species, because, except for mouflons (*4*), European wild ruminants generally are asymptomatic hosts. The highest peak of stress occurs during the mating period (August–September in Spain), which is also the period of maximal activity for *Culicoides imicola* mosquitoes. Therefore, all of these facts, together with the capability of wild ruminants to overcome BT infection and their free-range life, make deer suitable for BTV maintenance. We hypothesize that 1) BTV RNA would be detectable in red deer even after its control in livestock by vaccination, and 2) the virus or specific antibodies would be detected in red deer early after its detection in livestock.

## The Study

The study site was a deer farm with ≈900 hinds, including 550 adult hinds and 350 yearling hinds. This farm is located in the Los Alcornocales Natural Park in the Cádiz Province (Andalucía, southern Spain; 36°17′N, 5°47′W), an area near the sea that is <500 m above sea level. Abundant wild red deer and moderate densities of roe deer (*Capreolus capreolus*) are present in the area.

Blood samples were collected by cervical puncture from 510 living farmed red deer, placed in sterile tubes containing EDTA, and frozen at –20°C. Samples from adult deer hinds (n = 160) were obtained on July 12 and 13, 2007; yearling stags (n = 350) were sampled on August 28, 2007.

We tested 200 serum samples by using a competitive viral protein 7 (VP7) ELISA (Institute Pourquier, Montpellier, France). The samples were analyzed in duplicate according to the manufacturer’s instructions.

After RNA extraction from 510 red deer blood samples, RNA was analyzed by using 4 reverse transcription–PCRs (RT-PCRs): 1) a group-specific RT-PCR detecting a conserved region within the BTV nonstructural protein (NS) 1 segment (*6*); 2) a BTV-1 serotype-specific RT-PCR (*7*); 3) a BTV-4 serotype-specific assay (*8*); and 4) a group-specific RT-PCR that detects epizootic hemorrhagic disease (EHD) (*9*). BTV-4 PCR was performed as a 1-step real-time RT-PCR, and BTV-1, EHD, and the group-specific assays were conducted as gel-based, 1-step RT-PCRs. Prevalence of BT antibodies and BTV-1 and BTV-4 RNA and confidence intervals for prevalence (binomial exact, Clopper-Pearson) were calculated by using Quatitative Parasitology 3.0 software (*10*).

**Figure 1:**
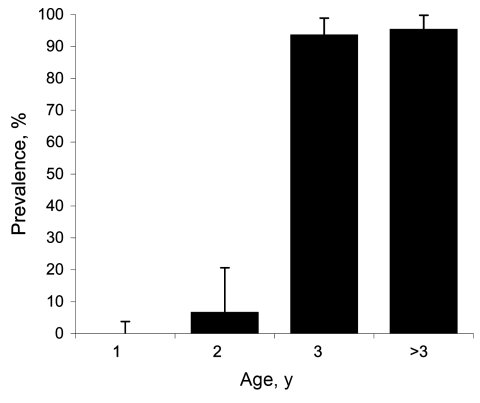
Results of ELISA to detect bluetongue virus (BTV) viral protein 7 in 200 serum samples collected from red deer, Spain. Results from yearlings were negative; results from adults showed an age-increasing trend of contact with BTV. Bars represent 95% confidence intervals for prevalence (binomial exact, Clopper-Pearson).

Of the analyzed serum samples, 57.60% showed positive results in the ELISA. The prevalence of BTV antibodies was high; 92.45% of the adults were positive. All yearling deer were ELISA negative except for 3 doubtful samples; all of them had negative results in the BTV, BTV-1 and BTV-4 RT-PCR assays (Figure 1).

Of the adult deer, 25% showed positive results in the BTV group-specific PCR. Positive samples were sequenced to confirm the presence of BTV nucleic acid and further analyzed for the identification of the serotype. Six RNA samples from adult deer were positive for the BTV-4–specific RT-PCR, and their sequences were confirmed by using BLAST software (http://blast.ncbi.nlm.nih.gov/Blast.cgi). None of the samples from adult deer were positive either for BTV-1-specific or EHD-specific RT-PCRs. Yearlings, however, showed a different pattern of results: 16.33% animals showed positive results in the group-specific and the BTV-1–specific RT-PCRs. No yearling samples were positive by the BTV-4 specific RT-PCR.

No visible clinical signs were noticed, and no deaths occurred. This result suggests that, although adult deer maintained circulation of BTV-4 RNA, this serotype did not infect the yearlings despite the presence of the vector and the optimal conditions for infection in the study area. Surprisingly, several animals were positive to the EHD-specific assay. However, when the PCR products were purified and sequenced, none of the obtained sequences showed homology with published EHD sequences. These results support those found by Agüero et al. (*11*), in which BTV-1–positive samples cross-reacted with the available EHD primers. The amplified PCR product obtained had approximately the same size as the PCR product expected for EHD, thus giving a false-positive result.

## Conclusions

Our results agree with what was found in livestock during surveillance programs: adult animals had probably been in contact with BTV-4 during the outbreak that started in southern Spain in 2004. In contrast to the vaccinated domestic ruminants, deer were able to maintain BTV-4 RNA, thus confirming our initial hypothesis. However, detection of BTV RNA without concurrent virus isolation does not mean that deer are a long term reservoir host of BTV (*12*). Simultaneous evaluation of adjacent cohorts of domestic and wild ruminants by using the same virus detection assays will be required to unambiguously define the precise role of wildlife in the epidemiology of BTV infection.

**Figure 2:**
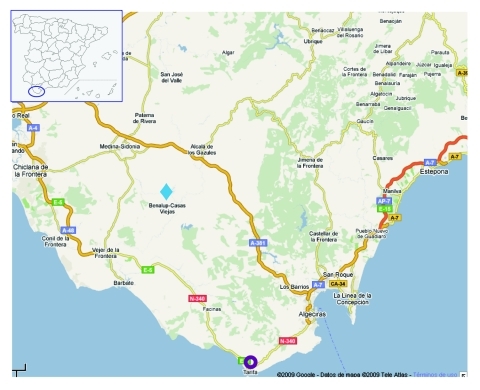
Epidemiologic situation for bluetongue virus (BTV) in Spain, July–August 2007. The first BTV-1 case in Spain was reported in Tarifa (purple circle), only 60 km west from a deer farm where the samples were collected (blue diamond). Map source: Google Maps.

Yearling deer were apparently infected with BTV-1, which has been present in Spain since 2007. When epidemiologic information about the study area was compared with the information for the deer samples analyzed, evidence was found supporting our results: adult deer were sampled on July 12, 2007, and yearlings were sampled August 20, 2007, i.e., 26 days after BTV-1 presence was confirmed at 60 km distance from the deer farm (www.oie.int/wahis/reports/en_imm_0000005799_20070726_123322.pdf) (Figure 2).

Thus, adult deer had been sampled when BTV-1 was not present in the country yet. In contrast, yearlings were already positive to BTV-1 only 26 days after this serotype was first reported in livestock in the same area. There are 2 explanations for this finding: 1) BTV-1 is a highly pathogenic serotype (*13*), causing high death rates in sheep, that may also cause high death rates in deer; and 2) deer and other wild ruminants may be highly susceptible to BTV infection, thus, making them good sentinels for this disease. However, BTV-1 was detected earlier among sentinel cattle than among deer.

Regarding EHD, despite the negative results obtained, lack of robust molecular tools for its detection is noteworthy. All available RT-PCRs are based on the sequences of EHD strains that have never been detected in the Mediterranean area.

## References

[1] Taylor WP. The epidemiology of bluetongue. Revue Scientifique et Technique de l'Office international des Epizooties. 1986; 5:351–6 10.20506/rst.5.2.256 32917067

[2] Ruiz-Fons F, Reyes-García AR, Alcaide V, Gortázar C. Spatial and temporal evolution of bluetongue virus in wild ruminants, Spain. Emerg Infect Dis. 2008;14:951–3. 10.3201/eid1406.071586 18507912 PMC2600300

[3] Linden A, Mousset B, Grégoire F, Hanrez D, Vandenbussche F, Vandemeulebroucke E, Bluetongue virus antibodies in wild red deer in southern Belgium. Vet Rec. 2008;162:459. 10.1136/vr.162.14.459-a 18390860

[4] Fernández-Pacheco P, Fernández-Pinero J, Agüero M, Jiménez-Clavero MA. Bluetongue virus serotype 1 in wild mouflons in Spain. Vet Rec. 2008;162:659–60. 18487587 10.1136/vr.162.20.659

[5] García I, Napp S, Casal J, Perea A, Allepuz A, Alba A, Bluetongue epidemiology in wild ruminants from southern Spain. Eur J Wildl Res. 2009;55:173–8. 10.1007/s10344-008-0231-6

[6] Agüero M, Arias M, Romero LJ, Zamora MJ, Sánchez-Vizcaíno JM. Molecular differentiation between NS1 gene of a field strain bluetongue virus serotype 2 (BTV-2) and NS1 gene of an attenuated BTV-2 vaccine. Vet Microbiol. 2002;86:337–41. 10.1016/S0378-1135(02)00011-1 11955783

[7] Mertens PP, Maan NS, Prasad G, Samuel AR, Shaw AE, Potgieter AC, Design of primers and use of RT-PCR assays for typing European bluetongue virus isolates: differentiation of field and vaccine strains. J Gen Virol. 2007;88:2811–23. 10.1099/vir.0.83023-0 17872535

[8] Rodríguez-Sánchez B, Iglesias-Martín I, Martínez-Avilés M, Sánchez-Vizcaíno JM. Orbiviruses in the Mediterranean basin: updated epidemiological situation of bluetongue and new methods for the detection of BTV serotype 4. Transbound Emerg Dis. 2008;55:205–14. 10.1111/j.1865-1682.2008.01029.x 18666964

[9] Ohashi S, Yoshida K, Yanase T, Kato T, Tsuda T. Simultaneous detection of bovine arboviruses using single-tube multiplex reverse transcription–polymerase chain reaction. J Virol Methods. 2004;120:79–85. 10.1016/j.jviromet.2004.04.006 15234812

[10] Rozsa L, Reiczigel J, Majoros G. Quantifying parasites in samples of hosts. J Parasitol. 2000;86:228–32. 10780537 10.1645/0022-3395(2000)086[0228:QPISOH]2.0.CO;2

[11] Agüero M, Buitrago D, Gómez-Tejedor C. False-positive results obtained when bluetongue virus serotype 1 Algeria 2006 was analyzed with a reverse transcription-PCR protocol for detection of epizootic hemorrhagic disease virus. J Clin Microbiol. 2008;46:3173–4. 10.1128/JCM.00353-08 18596148 PMC2546713

[12] MacLachlan NJ, Nunamaker RA, Katz JB, Sawyer MM, Akita GY, Osburn BI, Detection of bluetongue virus in the blood of inoculated calves: comparison of virus isolation, PCR assay, and in vitro feeding of Culicoides variipennis. Arch Virol. 1994;136:1–8. 10.1007/BF01538812 8002778

[13] Saegerman C, Berkvens D, Mellor PS. Bluetongue epidemiology in the European Union. Emerg Infect Dis. 2008;14:539–44. 10.3201/eid1404.071441 18394269 PMC2570923

